# Detection of Potential Arbovirus Infections and Pregnancy Complications in Pregnant Women in Jamaica Using a Smartphone App (ZIKApp): Pilot Evaluation Study

**DOI:** 10.2196/34423

**Published:** 2022-07-27

**Authors:** Elisa Ruiz-Burga, Patricia Bruijning-Verhagen, Paulette Palmer, Annalisa Sandcroft, Georgina Fernandes, Marieke de Hoog, Lenroy Bryan, Russell Pierre, Heather Bailey, Carlo Giaquinto, Claire Thorne, Celia D C Christie, ZIKAction Consortium

**Affiliations:** 1 Population, Policy & Practice Research and Teaching Department Great Ormond Street Institute of Child Health University College London London United Kingdom; 2 Julius Center for Health Sciences and Primary Care University Medical Center Utrecht Utrecht University Utrecht Netherlands; 3 Department of Child and Adolescent Health University of the West Indies Kingston Jamaica; 4 University Medical Center Utrecht Utrecht University Utrecht Netherlands; 5 Department of Obstetrics and Gynaecology University of the West Indies Kingston Jamaica; 6 Institute for Global Health University College London London United Kingdom; 7 Dipartimento di Salute della Donna e del Bambino Università degli Studi di Padova Padova Italy; 8 Fondazione Penta Onlus Padova Italy

**Keywords:** mHealth, digital health, arbovirus, pregnancy, adherence, compliance, low- and middle-income countries, LMIC, maternal health, pregnancy complications, prenatal care, pregnancy outcomes, mobile phone

## Abstract

**Background:**

There is growing evidence of the benefits of mobile health technology, which include symptom tracking apps for research, surveillance, and prevention. No study has yet addressed arbovirus symptom tracking in pregnancy.

**Objective:**

This study aimed to evaluate the use of a smartphone app (*ZIKApp*) to self-report arbovirus symptoms and pregnancy complications and to assess compliance with daily symptom diaries during pregnancy in a cohort of women in an arbovirus-endemic, subtropical, middle-income country (Jamaica).

**Methods:**

Pregnant women aged ≥16 years, having a smartphone, and planning on giving birth at the recruiting center were enrolled between February 2020 and July 2020. ZIKApp comprised a daily symptom diary based on algorithms to identify potential episodes of arbovirus infection and pregnancy complications. Sociodemographic, epidemiological, and obstetric information was collected at enrollment, with additional review of medical records, and users’ perception was collected through an exit survey. Descriptive analyses and logistic regression analysis of possible factors associated with diary adherence were performed.

**Results:**

Of the 173 women enrolled, 157 (90.8%) used ZIKApp for a median duration of 155 (IQR 127-173) days until pregnancy end, 6 (3.5%) used the app for <7 days, and 10 (5.8%) exited the study early. For each successive 30-day period from enrollment up to 150 days after enrollment, of these 157 women, 121 (77.1%) to 129 (82.2%) completed their daily symptom diary; 50 (31.8%) to 56 (35.7%) did so on the same day. Overall, 31.8% (50/157) of the women had *good adherence* to diary reporting (ie, they completed the task on the same day or 2 to 3 days later for ≥80% of the days enrolled). There were 3-fold higher odds of good adherence for participants aged >34 years versus those aged 25 to 29 years (adjusted odds ratio 3.14, 95% CI 1.10-8.98) and 2-fold higher odds for women with tertiary versus secondary education (adjusted odds ratio 2.26, 95% CI 1.06-4.83). Of the 161 women who ever made a diary entry, 5454 individual symptom reports were made (median 17 per woman; IQR 4-42; range 0-278); 9 (5.6%) women reported symptom combinations triggering a *potential arbovirus episode* (none had an adverse pregnancy outcome) and 55 (34.2%) reported painful uterine contractions or vaginal bleeding, mainly in the month before delivery. Overall, 51.8% (71/137) of the women rated the app as an excellent experience and were less likely to be poor diary adherers (*P*=.04) and 99.3% (138/139) reported that the app was easy to understand and use.

**Conclusions:**

This pilot found a high adherence to ZIKApp. It demonstrated the feasibility and usability of the app in an arbovirus-endemic region, supporting its future development to contribute to surveillance and diagnosis of arbovirus infections in pregnancy and to optimize maternal care.

## Introduction

### Background

Zika virus (ZIKV), chikungunya virus (CHIKV), and dengue virus (DENV) are arboviruses transmitted primarily by the *Aedes aegypti* mosquito that have caused multiple epidemics in recent years, notably the explosive epidemic of CHIKV in Latin America and the Caribbean that started in 2013, followed by the re-emergence of ZIKV a year later [1,2]. These arboviruses are of major public health concern and have been associated with significant morbidity alongside substantial economic impacts [3-5]. Although ZIKV outbreaks in particular have been highlighted with respect to maternal and infant health, owing to the causal link between ZIKV and microcephaly and other fetal and infant abnormalities [6], CHIKV and DENV are also both vertically transmitted, and all 3 viruses have been associated with adverse pregnancy outcomes (eg, preterm delivery, miscarriage, and stillbirth) and infant sequelae in the context of congenital infection [7-10].

Jamaica has experienced several DENV outbreaks of increasing intensity, severity, and magnitude in recent years (2010, 2012, and 2018-2019), with increased attributable morbidity and mortality in the very young, including reported cases of neonatal microcephaly [11-14]. Jamaica also experienced an explosive CHIKV epidemic in 2014 [3,15-18]: >80% of the general and antenatal populations were affected, with significant perinatal (maternal and newborn) illnesses, increased attributable neonatal morbidity and mortality [3,16,19], and >2500 deaths during the epidemic year nationally [18].

The ZIKV epidemic in Jamaica occurred in 2016-2017 [20-23]. National hospital-based surveillance revealed increased annual rates of severe microcephaly from 23.6 per 100,000 live births in 2010 (during the period of DENV outbreaks) to 41.7 per 100,000 live births in 2017 in association with the ZIKV epidemic [14], alongside surveillance reports of the congenital syndrome associated with ZIKV and related fetal brain disruption sequences temporally and spatially related to reported ZIKV cases [14]. The congenital syndrome was also being recognized simultaneously in 0.8% to 2.2% of the newborns in 3 urban public maternity hospitals [23], with a 15.6% ZIKV immunoglobulin G antibody seroprevalence in pregnancy reported [19]. Other complications included Guillain-Barré syndrome and varied neurological presentations in children and adults [21,22].

The COVID-19 pandemic has seen a growing number of symptom tracker mobile phone apps that have helped to develop an understanding of an emerging infection and its associated clinical manifestations [24-27]. Before the emergence of SARS-CoV-2, there was already an increasing body of research demonstrating the benefits of mobile health (mHealth) technology for remote monitoring of symptoms, public health surveillance, education, and prevention [28], including for arboviruses [29-32]. With respect to pregnancy, mHealth apps have been used for multiple interventions, including to optimize gestational weight gain, to increase intake of vegetables and fruit, for smoking cessation, to identify specific symptoms of pre-eclampsia, for drug safety monitoring, and to support health care delivery for prevention of asthma and infections [33-38]. However, there is limited literature on the levels of adherence to mHealth apps for daily reporting of symptoms during pregnancy. This is a significant gap in evidence because the requirement for daily reporting could be an important factor potentially limiting long-term adherence.

### Objectives

This pilot study aimed to evaluate the use of ZIKApp by pregnant women to report symptoms indicative of an arbovirus infection and pregnancy complications, including evaluating adherence with daily symptom diary reporting up to the end of pregnancy. The study setting was Jamaica, an example of an arbovirus-endemic, subtropical, middle-income country with high levels of smartphone penetration.

## Methods

### Recruitment

This pilot study was conducted at the University Hospital of the West Indies (UHWI), a university teaching hospital that performed approximately 1500 deliveries in 2016, as part of research conducted by the ZIKAction consortium, which conducts maternal and child health–focused research on ZIKV and other arboviruses in Latin America and the Caribbean. Participants were recruited between February 2020 and July 2020 from the antenatal care service by research nurses. This pilot study sought to enroll approximately 200 participants who met the following eligibility criteria: pregnant women aged ≥16 years who planned to give birth at the UHWI and had access to a smartphone compatible with the mobile app. Following these criteria, the research nurses orally explained the study, invited these pregnant women in the waiting room of this antenatal clinic on their first medical clinic visit (when the general educational talks are delivered) to take part in the study, and enrolled those interested. Furthermore, some of the participants also told other pregnant women about the study and recommended their participation, after which these women would also individually approach the research nurses, who would follow-up with the standard enrollment procedures. Names and contact details were stored locally by the research nurses, but all study questionnaires were pseudonymized with the use of unique study identifiers. Enrollment visit procedures are presented in the *Study Implementation* section.

### The ZIKApp Intervention

**Figure 1:**
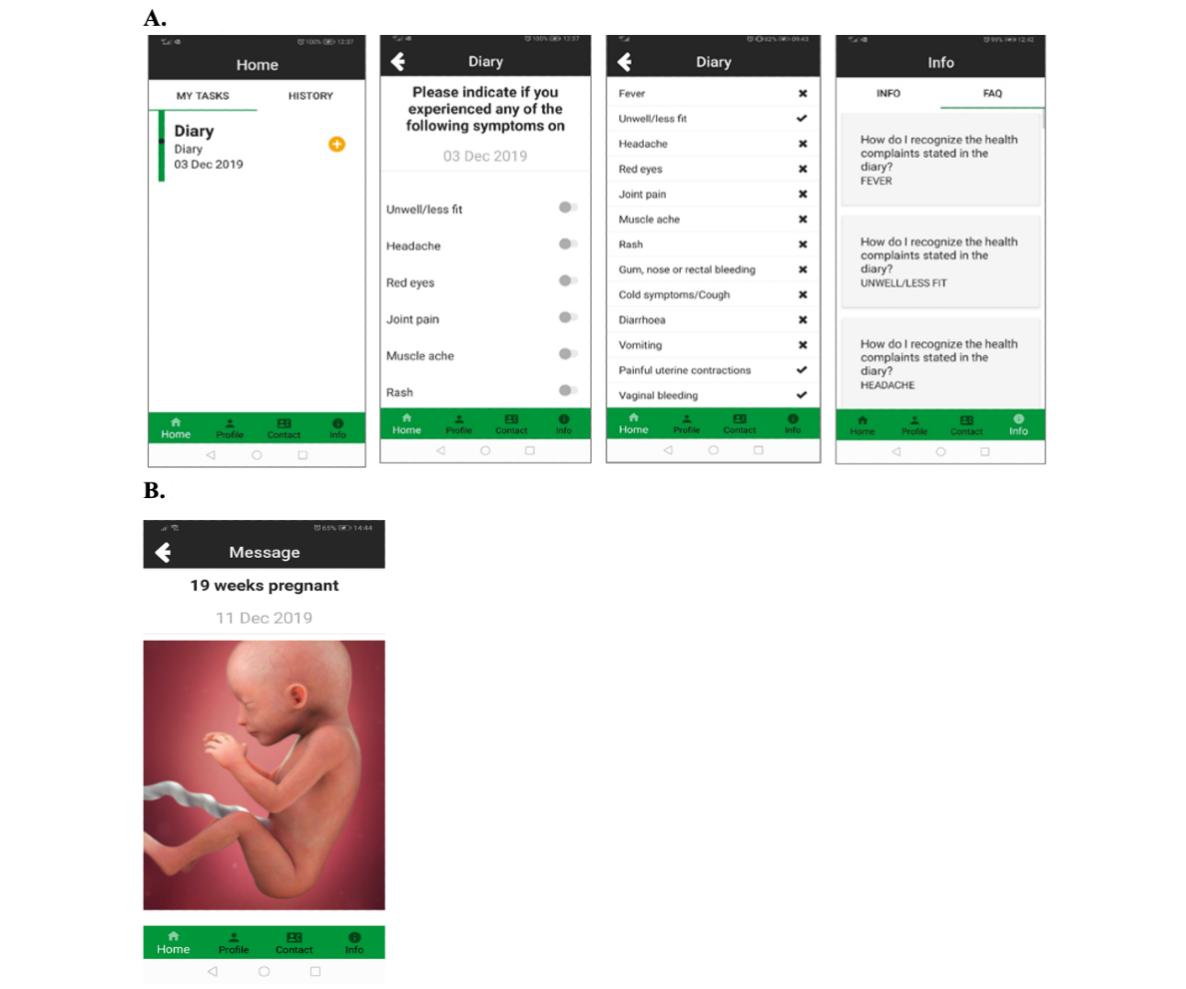
ZIKApp screenshots. (A) Diary and symptom information. (B) Example of an informative message regarding pregnancy: “...your baby at week 19. The top of your womb (uterus) now reaches your belly button and will grow about a centimetre higher than this each week. Your baby measures up to 15.3 cm (6 in) from head to bottom (crown to rump) and weighs about 240 g (8.5 oz). He’s the size of a big tomato...”.

ZIKApp was developed by the University Medical Center Utrecht in partnership with Your Research, a company based in the Netherlands. ZIKApp is compatible with both Android and iOS platforms. The app included a daily symptom diary for reporting presence or absence of symptoms and provided users with information on potential symptoms. In addition, the app provided information about the study itself and included a *Frequently Asked Questions* section (Figure 1A).

The app was designed to provide regular informative messages related to the pregnancy such as “Your baby is now the size of a pear,” similar to content provided by commercial pregnancy tracking apps (Figure 1B). This was to provide an additional incentive for women to adhere to app use.

Participants were sent daily reminders by email to complete their symptom diary. The daily diary entry could be completed on the same day or with a lag period of up to 7 days, after which it was no longer accessible. The symptoms included in the app and the start and end of arbovirus episode triggers are presented in Multimedia Appendix 1 and were selected to identify symptoms suggestive of arbovirus infection as well as 2 specific pregnancy complications (painful uterine contractions and vaginal bleeding). The app also included some short periodic questionnaires; for example, the first questionnaire requested information on the woman’s last menstrual period date (to allow the app to send gestation-appropriate messages). Data recorded in the app were stored on a secure cloud-based portal (ResearchFollowApp portal). The portal also provided a user-friendly dashboard for the research nurses with an authorized log-in to monitor in real time the symptoms reported by participants.

### Study Implementation

A standard operating procedure (SOP) was developed to guide the research nurses, and in-person training was conducted at the UHWI in January 2020. The SOP detailed the different tasks that research nurses should fulfill during the recruitment once informed consent was obtained. It explained the process of setting up an account for the participant through the portal, assisting her in downloading the app from the Google Play Store or Apple App Store, guiding the initial app log-in, instructing her about the different app functions and notifications that she could receive (*potential arbovirus episode* or *possible pregnancy complication*), and guiding her to complete her first diary entry. Likewise, the SOP detailed the process to report any app or network issues to the coordinating University College London and University Medical Center Utrecht teams to resolve them and thus avoid early exits by participants and to improve app functionality.

At recruitment, a standardized form was used to collect additional sociodemographic, obstetric, and clinical information regarding the participant extracted from the medical chart. Data were entered and managed using REDCap (Research Electronic Data Capture; Vanderbilt University) hosted at Penta Foundation ONLUS [39,40]. REDCap is a secure, web-based software platform designed to support data capture for research studies. The ResearchFollowApp portal was also designed to facilitate the follow-up of participants by the research nurses and to send automatic emails and notifications. The research nurses implemented the use of WhatsApp messages to inform participants about their office hours and to advise those who received notifications of *potential arbovirus episode* or *possible pregnancy complication* to contact the health services using a provided list of phone numbers. All participants received monthly phone credits to enable internet access to allow the app to transmit the data collected to the portal.

Study participation ended when the participant gave birth or when the pregnancy came to an end for other reasons (eg, miscarriage), although the women could withdraw from the study at any time. At study exit, the research nurses guided participants to uninstall ZIKApp and invited them to complete an exit survey that included questions on their experience of using the app. In addition, a standardized questionnaire was used to collect data regarding the delivery as well as details of the newborn, which were entered in the study REDCap database. A case note review was conducted for women with potential arbovirus triggers to identify any maternal diagnoses (eg, from the laboratory information systems). During the pilot study, surveillance for SARS-CoV-2 among the pregnant women was implemented at the UHWI, and a case note review was also carried out for enrolled women with positive SARS-CoV-2 tests.

### Statistical Analysis

App data stored on the ResearchFollowApp portal was downloaded and merged with data from the REDCap database using unique study identifiers before analysis. Descriptive analyses of participant characteristics were conducted. Univariable comparisons of categorical variables were assessed using chi-square or Fisher exact tests. To assess participants’ adherence to completing the daily symptom diary, every diary day for each woman (ie, for the total time they used the app) was coded into 1 of 4 categories: diary completed on the same day, 2 to 3 days later, 4 to 7 days later, and >7 days elapsed without diary entry (ie, not completed). Next, for each woman, 2 binary variables (0 and 1) were created: for *good adherence* and *poor adherence* for their entire period of enrollment. *Good adherence* was where a participant had completed her diary on the same day or 2 to 3 days later for at least 80% of the time between enrollment and pregnancy end. *Poor adherence* was where there was an uncompleted diary (ie, >7 days had elapsed without diary entry) 30% of the time.

Potential factors associated with *good* and *poor* adherence were assessed using logistic regression analysis to obtain the odds ratios with 95% CIs: participant age, education, number of children, having an income, previous adverse pregnancy outcome, comorbidities and chronic diseases, duration of app use, and whether a potential arbovirus episode was reported. Several factors were considered when selecting the final multivariable models: first, all variables that were significant with a *P* value of <.10 in univariable analysis were considered for inclusion; second, a backward stepwise selection approach was used to determine the final adjusted model. Stata software (version 16.1; StataCorp LLC) was used to conduct the analyses.

### Ethics Approval

The protocol for this study was reviewed and approved by the University College London Research Committee on 27 September 2021 (Project ID 3715/005) and by the University of the West Indies Mona Campus Research Ethics Committee (project ID ECP 47, 19/20). All participants signed an informed consent form during the enrollment.

## Results

### Overview

**Figure 2:**
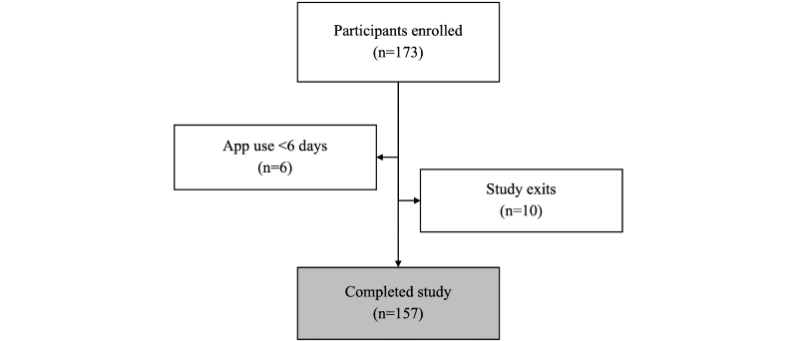
Study participant flow chart.

A total of 173 pregnant women were enrolled in the study (Figure 2), with the last delivery occurring on January 5, 2021. Of these 173 women, 5 (2.9%) had no data recorded in the app portal, indicating that they never used the app (although, of these 5 women, for 2, 40%, this may have been a technical or connection issue as both reported completing the diary in their exit survey) and 1 (0.6%) had <1 week of study participation owing to a miscarriage 6 days after enrollment. These women were excluded from further analyses. Of the remaining 167 women, 157 (94%) used the app until they gave birth or the end of their pregnancy, whereas 10 (6%) chose to exit the study before they gave birth.

Table 1 presents the participants’ baseline characteristics, stratified by study completion and study exit status. Overall, of the 167 women, 166 (99.4%) were born in Jamaica and 1 (0.6%) was born in the United Kingdom; most (137/167, 82%) of the women were enrolled in the second trimester of pregnancy. Of the 10 women who exited the study, 6 (60%) gave phone-related reasons for their study exit (eg, no longer having a smartphone or having changed their phone), 2 (20%) changed their mind about study participation, and 2 (20%) cited problems with phone credit reimbursement. There were no statistically significant differences between the women who exited the study and those who remained and used the app until they gave birth with respect to sociodemographic characteristics and comorbidities (data not shown).

**Table 1:** Participant sociodemographic, clinical, and app use characteristics by study completion and study exit status (N=167).

Characteristics			Completed study (n=157)		Exited study (n=10)
Age (years), median (IQR; range)			28 (24-32; 18-44)		26.5 (22-31; 21-36)
**Marital status, n (%)**					
	Married	45 (28.7)		1 (10)	
	Cohabiting	47 (29.9)		3 (30)	
	Single	60 (38.2)		6 (60)	
	Divorced or separated	5 (3.2)		0 (0)	
**Highest level of education, n (%)**					
	Secondary	61 (38.9)		3 (30)	
	Tertiary	96 (61.2)		7 (70)	
**Employed or has regular income, n (%)**					
	No	77 (49)		3 (30)	
	Yes	80 (51)		7 (70)	
**Parity, n (%)**					
	Nulliparous	90 (57.3)		5 (50)	
	Primiparous	41 (26.1)		3 (30)	
	Multiparous	26 (16.6)		2 (20)	
**Chronic conditions, n (%)**					
	None	93 (59.2)		6 (60)	
	Hypertension^a^	26 (16.6)		0 (0)	
	Sickle cell disease^a^	11 (7)		0 (0)	
	Pre-existing or gestational diabetes^a^	7 (4.6)		0 (0)	
	Asthma^a^	24 (15.3)		1 (10)	
	Obesity^a^	6 (3.8)		1 (10)	
Gestational age at enrollment (weeks), median (IQR; range)			18 (15-22; 11-38)		17 (16-18; 14-21)
**Pregnancy outcome, n (%)**					
	Live birth	151 (96.2)		N/A^b^	
	Stillbirth	2 (1.3)		N/A	
	Miscarriage	3 (1.9)		N/A	
	Termination (abnormality)	1 (0.6)		N/A	
**Gestational age at delivery^c^ (weeks), n (%)**					
	<34	4 (2.7)		N/A	
	34 to 36	10 (6.6)		N/A	
	≥37	136 (90.1)		N/A	
Duration of app use (days), median (IQR; range)			155 (127-173; 25-235)		133 (104-172; 80-179)

^a^Multiple responses were possible.

^b^N/A: not applicable.

^c^Live births only (1 unknown).

### Daily Symptom Diary Reporting and Factors Associated With Good and Poor Adherence

**Figure 3:**
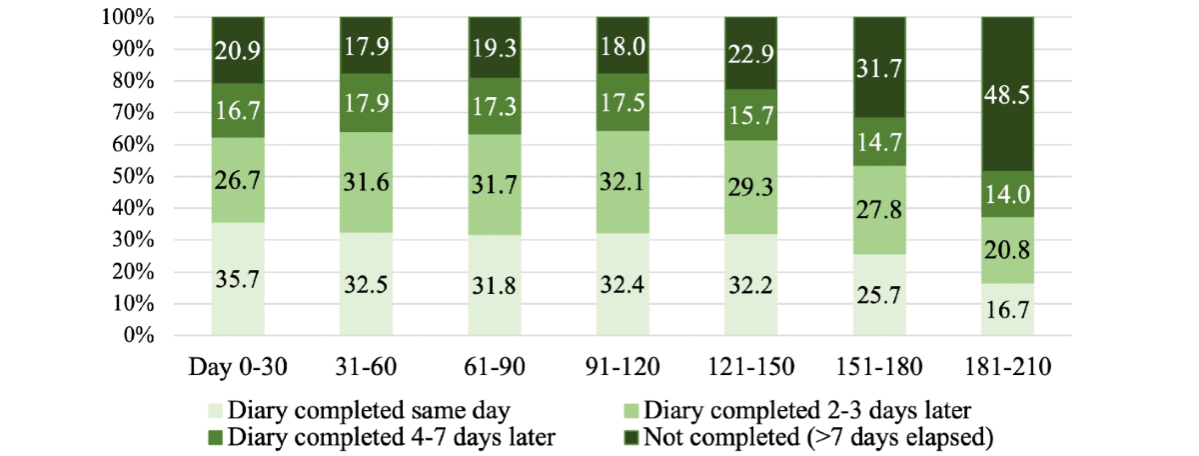
Timing of symptom diary completion, by 30 day period from initiation of app use (n=157). The raw data corresponding to the percentages, and the number of women contributing to each 30 day period, are shown in Multimedia Appendix 2.

Overall, 78.68% (17,833/22,664) of the daily diaries were completed over the study period. The timing of symptom diary completion (ie, same day, 2-3 days later, 4-7 days later, or not completed) per successive 30-day periods between enrollment and pregnancy end is presented in Figure 3 and Multimedia Appendix 2. For each of these periods (up to 150 days), of these 157 women, between 121 (77.1%) and 129 (82.2%) completed their daily symptom diary, with the proportion of diaries completed on the same day per 30-day period (up to 150 days) staying roughly constant at one-third (Figure 3; Multimedia Appendix 2).

The proportion of noncompleted diaries (ie, >7 days elapsed without diary completion) increased from the fifth month of app use. From day 181 onwards, the 7-day period within which a participant could complete her symptom diary retrospectively would have likely encompassed the date of delivery. Considering only diary days that were completed, there was also evidence of a reduced timeliness of reporting with increasing enrollment duration, with 41.69% (1024/2456), 37.67% (440/1168), and 32.4% (56/173) of the diaries completed on the same day at days 121-150, 151-180, and 181-210, respectively (*P*<.001).

To understand whether external events (eg, the Christmas holiday season) had an impact on the timeliness of diary completion, we compared the period from December 19, 2020, to January 2, 2021, and the period from December 1, 2020, to December 15, 2020, and found that the proportion of diaries completed on the same day was higher in the earlier period than during the Christmas period at 33.5% (190/568) versus 23.1% (78/337), respectively, although the proportion of noncompleted diaries was lower at 24.6% (140/568) versus 29.4% (99/337; *P*=.01), respectively.

Among the 157 women who used the app until the end of their pregnancy, there were 50 (31.9%) classified as having good adherence to diary completion (ie, completed same day or 2-3 days later at least 80% of the days). In univariable analysis (Table 2), women in the oldest age group (women aged >34 years vs those aged 25-29 years) and with tertiary education level (vs secondary education level) had higher odds of good adherence, although there was no association between good adherence and history of adverse pregnancy outcome, having a regular income, number of children, chronic disease status, duration of app use, or report of symptoms of arbovirus infection through the app. In the adjusted model (which included age and education only), both variables remained independently associated with good adherence, with the odds of good adherence being 3-fold higher for participants aged >34 years compared with those aged 25 to 29 years and 2-fold higher for women with a tertiary education compared with those who received a secondary education (Table 2).

In total, 24.8% (39/157) of the participants were classified as poor adherers to the symptom diary. Consistent with findings for good adherence, older women (those aged >34 years) had a significantly lower odds (adjusted odds ratio 0.10, 95% CI 0.01-0.80) of being poor adherers than participants aged 25 to 29 years. In addition, a short duration of app use (ie, <90 days) was associated with poor adherence (adjusted odds ratio 2.71, 95% CI 1.06-6.93).

**Table 2:** Factors associated with good adherence to symptom diary reporting (N=157).

Explanatory variables			Values, n (%)		Good adherence, n (%)		Unadjusted OR^a^ (95% CI)		*P* value		Adjusted OR (95% CI)		*P* value
**Participant age (years)**													
	25 to 29	53 (33.8)		15 (28.3)		Reference		N/A^b^		Reference		N/A	
	<25	40 (25.5)		10 (25)		0.84 (0.33-2.15)		.72		0.97 (0.37-2.51)		.95	
	30 to 34	42 (26.8)		13 (31)		1.14 (0.47-2.75)		.78		1.22 (0.49-3.00)		.67	
	>34	22 (14)		12 (54.6)		3.04 (1.09-8.52)		.03		3.14 (1.10-8.98)		.03	
**Previous adverse pregnancy outcome**													
	No	110 (70.1)		35 (31.8)		Reference		N/A		N/A		N/A	
	Yes	47 (29.9)		15 (31.9)		1.00 (0.48-2.09)		.99		N/A		N/A	
**Regular income**													
	No	77 (49)		23 (29.9)		Reference		N/A		N/A		N/A	
	Yes	80 (51)		27 (33.8)		1.20 (0.61-2.34)		.60		N/A		N/A	
**Number of children**													
	0	90 (57.3)		26 (28.9)		Reference		N/A		N/A		N/A	
	1	41 (26.1)		14 (34.2)		1.28 (0.58-2.81)		.55		N/A		N/A	
	>1	26 (16.6)		10 (38.5)		1.54 (0.62-3.83)		.36		N/A		N/A	
**Education**													
	Secondary	61 (38.9)		13 (21.3)		Reference		N/A		Reference		N/A	
	Tertiary	96 (65.3)		37 (38.5)		2.32 (1.11-4.84)		.03		2.26 (1.06-4.83)		.03	
**Comorbidities and chronic diseases**													
	No	93 (59.2)		29 (31.2)		Reference		N/A		N/A		N/A	
	Yes	64 (40.8)		21 (32.8)		1.08 (0.55-2.13)		.83		N/A		N/A	
**Duration of app use (days)**													
	<90	25 (15.9)		7 (28)		Reference		N/A		N/A		N/A	
	≥90	132 (84.1)		43 (32.6)		1.24 (0.48-3.20)		.65		N/A		N/A	
**Symptoms of arbovirus infection reported**													
	No	148 (94.3)		49 (33.1)		Reference		N/A		N/A		N/A	
	Yes	9 (5.7)		1 (11.1)		0.25 (0.03-2.08)		.20		N/A		N/A	

^a^OR: odds ratio.

^b^N/A: not applicable.

### Reporting of Symptoms Overall

**Figure 4:**
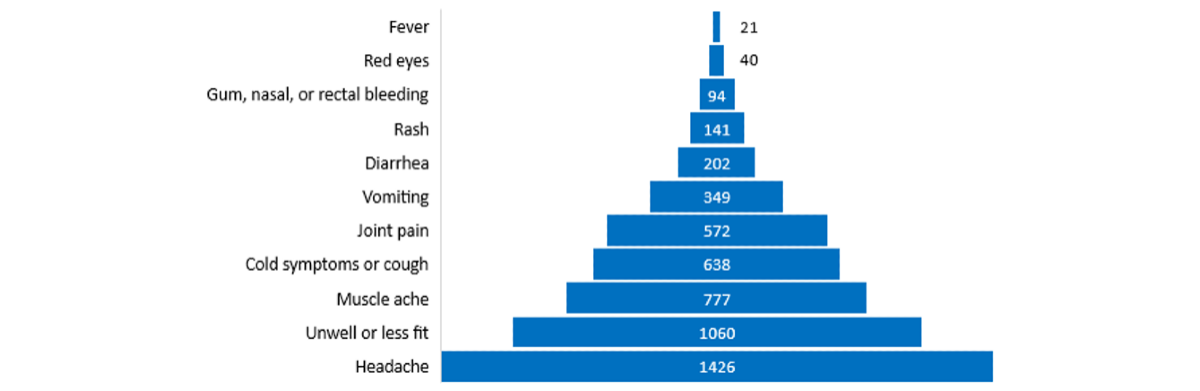
Count of total reports of symptoms (excluding pregnancy complications).

Overall, across the 17,883 completed diaries, there were 5454 (30.5%) individual reports of symptoms (n=5320, 29.75%, if vaginal bleeding and painful uterine contractions were excluded), including symptoms that may have been reported on the same day (eg, a headache and cold or cough). The most commonly reported symptoms were headache and feeling unwell or less fit (Figure 4). Of the 161 women who ever made a symptom diary entry (including n=4, 2.5%, who exited the study early), the median number of symptoms reported per woman was 17 (IQR 4-42; range 0-278); 17 (10.6%) women never reported a symptom.

### Potential Arbovirus Infection Symptom Episodes

In total, 5.6% (9/161) of the participants reported a combination of symptoms that triggered a *potential arbovirus episode*, with 1 episode each at a median 26 (range 13-36) gestational weeks. The distribution of symptoms for these women, including those reported just before and after the episode, is presented in Multimedia Appendix 3. Of these 9 women, 4 (44%) reported some symptoms (headache, joint pain, or rash) the day before the notification of a potential arbovirus episode was triggered. In addition, of the 9 women, 2 (22%) had patterns of symptoms that resulted in both type 1 and 2 triggers and both reported additional symptoms (vomiting and cold symptoms or cough). Of the remaining 7 women, 5 (71%) reported one or more symptoms in the episode in addition to the relevant trigger symptoms, with cold symptoms or cough being the most common (reported by n=3, 43%; Multimedia Appendix 3).

No diagnosis of an arbovirus-related illness in relation to these episodes was made for any of the women (n=7) where detailed chart reviews were conducted. The women kept their regular antenatal appointments and had abdominopelvic ultrasound scans (anomaly scan and usually one more) to evaluate maternofetal health throughout pregnancy (which is the policy for evaluating any complications of arbovirus-related illness). Many (6/7, 86%) of the women had intercurrent illnesses in pregnancy, including cholestasis, urinary tract infections (several), ligamentous pain, otitis media, gastroesophageal reflux disease, and pre-eclampsia. All the women (n=9) delivered live births. None had SARS-CoV-2 detected in surveillance swabs.

### COVID-19 Diagnoses

Of the 157 participants, 3 (1.9%) had laboratory-confirmed SARS-CoV-2 infection (nasopharyngeal swab positive for SARS-CoV-2 by polymerase chain reaction; all gave birth in late September and mid-October 2020). Of these 3 women, 1 (33%) reported headache over a 3-day period through the app, starting 8 days before the diagnosis; the case note review indicated additional symptoms not reported in the symptom diary (fever and cough), including anosmia and ageusia (not possible to report through the app); 1 (33%) reported headache on the day of the SARS-CoV-2 diagnosis and then 3 separate episodes of joint pain in the 10 days after the diagnosis; there was no record of these symptoms in her medical notes; and 1 (33%) reported feeling unwell or less fit for a 2-day period starting 4 days before the SARS-CoV-2 positive result; no symptoms were recorded in her medical notes.

### Potential Pregnancy Complication Episodes

Of the 157 participants, 55 (35%) reported 114 pregnancy complication symptoms (painful uterine contractions or vaginal bleeding) up to, and including, the day of delivery or the end of pregnancy. Delivery occurred at term in 85% (47/55) of the women, at 34 to 36 weeks’ gestation in 7% (4/55), and before 34 weeks in 4% (2/55). Of the 55 women, 2 (4%), miscarried. Among the 114 pregnancy complication symptoms, there were 59 (51.8%) reports of painful uterine contractions, which occurred at a median of 36 (IQR 5-108; range 0-199) days before delivery, and 39 (34.2%) reports of vaginal bleeding, which occurred at a median of 64 (IQR 6-127; range 0-174) days before delivery. Nearly a third of these symptom reports occurred within 7 days before delivery (35/114, 30.7%, for both). There were 8 episodes where both these symptoms were reported on the same day, in all cases in the 3 days before delivery.

### Adverse Pregnancy Outcomes and Symptom Reporting

Of the 5 participants whose pregnancy ended in miscarriage or stillbirth, 2 (40%) did not complete their diary in the 2 weeks before the end of the pregnancy. Of the remaining 3 participants, 2 (67%) reported pregnancy complications during this period; in addition, all (3/3, 100%) reported one or more of the following symptoms: unwell or less fit, rash, headache, red eyes, muscle ache, cold symptoms or cough, and diarrhea (but without activating the potential arbovirus trigger).

### Participants’ Perceptions of the App

Of the 157 participants who used the app until delivery or the end of their pregnancy, 139 (89%) participated in the exit survey. Regarding their experience of participating in the study, 51.8% (71/137) rated it as excellent (5 on a scale of 1-5), with 35.8% (49/137), 11.7% (16/137), and 0% (0/137) giving ratings of 4, 3, and 2, respectively. Of the 137 respondents, only 1 (0.7%) reported her experience as disappointing (rating of 1). The women who reported an *excellent* experience were less likely to be poor adherers to symptom diary completion than other women, with 18% (13/71) having poor adherence compared with 33% (22/66) who had good adherence (*P*=.04).

Of the 139 respondents, 138 (99.3%) reported that the app was easy to understand and use. Overall, 52.5% (73/139) of the women reported experiencing technical difficulties while using the app (ie, difficulties accessing Wi-Fi to complete their diary, difficulties accessing the internet on their mobile phone, and app freezing). However, experiencing technical difficulties was not associated with poor adherence (*P*=.90) or good adherence (*P*=.60; data not shown).

Of the 10 women who exited the study before delivery, 5 (50%) participated in the exit survey. With respect to their experience as participants in the study, of the 5 respondents, 2 (40%) rated their experience as excellent, 2 (40%) gave a rating of 4, and 1 (20%) gave a rating of 2. Of these 5 women, 5 (100%) reported that the app was easy to understand and 4 (80%) reported that it was easy to use. In addition, of the 4 women who answered the section on technical difficulties, 2 (50%) reported experiencing them while using the app.

## Discussion

### Principal Findings

This pilot study enrolled 173 pregnant women attending antenatal care at a university hospital clinic in Jamaica to evaluate their longitudinal engagement with a smartphone app to report symptoms potentially associated with arbovirus infections and pregnancy complications. Specifically, we wanted to understand whether it was feasible for pregnant women to report through the app the presence or absence of symptoms as well as symptom type on a daily basis over a prolonged period (from the second trimester to delivery).

This 1-year pilot study achieved a larger sample than pilot studies of other apps in pregnancy, for example, monitoring weight gain (2 studies recruiting ≤100 women [34,41]) or on reducing stress (29 women) [42], which is noteworthy given that our pilot overlapped with the beginning of the COVID-19 pandemic that forced many countries to stop or reduce ambulatory health care. We found that very few (10/173, 5.8%) women exited the study early and only 3.5% (6/173) never used the app after enrollment. This compares favorably with a study in Germany, which involved monthly web-based visits and surveys to digitally assess pregnancy-related symptoms and complications (including physical symptoms, depression, and anxiety), in which 7% of the women formally exited the study and 55% overall stopped using the app and made no further contact with the study team [43]. Thus, initial concerns that there might be a high rate of attrition because of the perceived burden of symptom reporting were not borne out in our pilot study.

ZIKApp was designed to *tolerate* delayed reporting of symptoms (ie, up to 7 days later), with the rationale that this cutoff would maximize reporting over the remainder of pregnancy while minimizing recall bias and allowing for any temporary problems with internet access. Adherence to symptom reporting through the app was good, with 79% (17,905/22,664) of all diaries completed. As shown in our figure of timing of symptom diary completion (Figure 3), overall patterns were fairly consistent with increasing duration of app use (considering successive 30-day periods) over the first 20 weeks after enrollment, with approximately a third of the diaries completed on the same day during this period (Multimedia Appendix 2). For the first 5 months of enrollment, only approximately 20% of the daily diaries were not completed at all (Multimedia Appendix 2).

However, patterns after 150 days of enrollment showed a decrease in diary completion overall, which may partly reflect women giving birth while they were within the 7-day window for daily diary completion. However, considering only those women who completed a diary, we showed that, as the duration of enrollment increased, there was a significant decline in the proportion completing their diary on the same day. The challenge of long-term adherence as a potential drawback of self-monitoring of symptoms through e-diaries has also been confirmed in other studies in pregnant and nonpregnant women that have reported less frequent diary reporting over calendar time [43-50].

A possible explanation for the good engagement of our participants could be the push notifications and reminders that were sent through our app as well as through email that could have been essential cues to action that encouraged our participants to complete their diaries or communicate with health care providers. This would be consistent with findings from a trial evaluating an mHealth intervention for healthy weight gain in pregnancy, which had good engagement in the intervention arm (only 9% attrition overall) in which SMS text messaging was a central feature [34]. The provision of phone credits in our study is also likely to have contributed because poor socioeconomic status has been associated with low engagement with an app for pregnant women elsewhere [51].

We found that older women (those aged >34 years) and women who had been to college or university were more likely to be good adherers to symptom diary reporting than younger women and those with less education, respectively. This finding supports other studies that have shown social variables such as older age and higher educational level to be significantly associated with sustained app use, including symptom diaries [43,44,47,52]. Other variables such as having a regular income, number of children, history of adverse pregnancy outcome, having a chronic condition, duration of app use, and reporting arbovirus infection symptoms were not found to be associated with good adherence in our study in contrast to others [44]. It was interesting to note that, although there was lower diary reporting overall and reduced timeliness of symptom reporting over the Christmas period (consistent with competing priorities during the busy holiday season), there was no association between other external factors such as technical difficulties using the app (eg, internet access and wireless connectivity) and adherence. In Jamaica, because the Christmas period is known to be a period of increased deliveries (including preterm births), the significant association with decreased app use is an important observation that should be considered for future implementation.

Of the 161 women who ever made a diary entry, 145 (90.1%) reported at least one symptom over a median of 22 weeks of app use. There was substantial heterogeneity in symptom reporting, with 1 in 10 women reporting no symptoms, whereas some (5/161, 3.1%) reported >200 symptoms. A potential challenge of using symptom diaries in pregnancy to identify signals of potential infections is the *noise* generated as a result of common pregnancy-related symptoms. Triggers were selected to try to differentiate between *noise* and signals of true infections, but the pilot was not designed to evaluate the diagnostic performance of these triggers, which would require a different type of study to include diagnostic follow-up of all women with and without episode triggers. The most common symptoms reported in our study were headache, feeling unwell or less fit, and muscle ache, whereas the relatively low frequency of vomiting may reflect the fact that the first quartile of gestational age at enrollment was 15 weeks. It should also be noted that 45.5% (76/167) of the participants had at least one chronic condition, although participation in the study per se could have meant that the women were more sensitized to any physical symptoms they were experiencing and reported accordingly, whereas the normal ailments of pregnancy may have been exaggerated in other women, especially in light of the ongoing COVID-19 pandemic, potentially creating additional stressors for the pregnant woman.

This pilot study coincided with the start of the COVID-19 pandemic in Jamaica and as the study progressed, national surveillance for SARS-CoV-2 moved from passive to active surveillance, with pregnant women screened for symptomatic illness and asymptomatic involvement. Simultaneously, an unlinked serosurvey was performed in this antenatal population, which showed increasing SARS-CoV-2 seroprevalence, from 6.9% in September 2020 to 16.9% in October 2020 and 24% in November 2020; of the 37 pregnant women who tested SARS-CoV-2 immunoglobulin G antibody positive, only 3 were symptomatic [53]. The app development predated the emergence of SARS-CoV-2 but did capture some symptoms commonly reported with COVID-19. It was interesting to note that of the 3 pregnant women diagnosed with SARS-CoV-2, 2 (67%) reported symptoms approximately at the time of infection through the app that were not reported in the medical notes, whereas 1 (33%) did not report the fever and cough she experienced through the app (but delivered preterm within 4 days of experiencing symptoms and 2 days of testing positive).

The findings of this pilot suggest that despite most arbovirus infections being asymptomatic, screening pregnant women for relevant symptoms can improve case detection among those who are symptomatic [54], and that was part of the rationale for the development of ZIKApp. Our perspective is supported by recent evidence showing that a simple score based on clinical data and laboratory results provides a useful tool to help diagnose arbovirus infections [55].

To improve the usefulness of the app, it would possibly be more valuable to implement it during an epidemic period, rather than during a period of low prevalence of circulating arboviruses in the community, which was the case during this study. We also incorporated 2 symptoms (painful vaginal contractions and vaginal bleeding) that could signal important pregnancy complications (depending on timing).

We obtained feedback from participants about their experience of using the app in the exit survey, which had a high participation (139/157, 88.5%). More than half (71/137, 51.8%) of the responding women stated that their experience in the study was excellent and, consistently, they were less likely to be poor adherers to diary completion. Almost all (138/139, 99.3%) reported that the app was easy understand and use, despite a relatively high proportion experiencing technical difficulties at some point. This finding differs from a study in pregnant women with gestational diabetes that found that technological problems with the app had a negative impact on user satisfaction [56]. Overall, our exit survey results corroborate other mHealth intervention results that show ease of use and simplicity [33,44,56] and ease of navigation and ease of understanding [57,58] are key features of apps that can influence sustained adherence. Furthermore, our findings support previous evidence about the role of the perception of the product on intended app use by women [59].

Our finding of *long and strong* adherence to symptom diary reporting in pregnant women in this Jamaican setting provides important evidence to inform the potential applications of apps where symptom diaries and self-monitoring may be used as a tool for research (eg, to develop a better understanding of patient-reported outcomes), for surveillance and participatory epidemiology (eg, as seen for tracking COVID-19 or influenza), or for clinical purposes (eg, for remote health monitoring of low-risk pregnancies, the importance of which was highlighted by the disruption of traditional pathways for health care during the COVID-19 pandemic). Our experience in this pilot study showed that future implementation of this intervention for clinical use will require women to be linked directly to clinical care providers in different health services (eg, antenatal clinic, labor ward, emergency department, high-risk obstetric ward, and newborn services) rather than through research nurses to allow interpretation of symptoms and provision of appropriate clinical care in real time.

### Limitations

We were unable to conduct the planned qualitative aspects of this study (ie, focus discussion groups with a sample of participants and staff) because of COVID-19–related restrictions, although we were able to obtain data on user perceptions through the exit survey. We were therefore unable to explore other potential facilitators (eg, the generic pregnancy-related messages embedded in the app, acceptability of answering questionnaires through the app, the role of the research nurses, and the perceived role of the app in the context of the COVID-19 pandemic) as well as barriers to app engagement (eg, whether feeling unwell had an impact on the timeliness of diary completion) or cues to action regarding health-seeking behaviors. Questions relating to facilitators and barriers to prolonged engagement with health-monitoring apps in pregnancy as well as linked health-related behaviors therefore require future research (eg, with mixed methods approaches).

Other limitations of this pilot study include the possibility of selection bias because only pregnant women who approached the nurses after the initial information provision in the clinic waiting room were recruited into the study. Consequently, our participants likely represent pregnant women using this antenatal clinic who were willing to take part in an mHealth intervention study. Likewise, women who were willing to participate were potentially more likely to adhere to diary reporting than nonparticipants, potentially resulting in social desirability response bias, particularly with respect to the exit survey. In addition, the results of this pilot study may not be generalizable to all pregnant women in Jamaica because of potential sociodemographic differences between our study population and the general antenatal population; for example, there were higher proportions of women who were nulliparous and who had received tertiary education in our study than in another ZIKAction consortium study that enrolled pregnant women from across the Kingston, Jamaica, metropolitan area [60]. Further research could recruit women from community-based antenatal clinics to capture a more socioeconomically diverse sample.

### Conclusions

We have demonstrated the feasibility and usability of ZIKApp in an arbovirus-endemic region, showing that most pregnant women were able to adhere to symptom reporting through the app for a prolonged period and supporting its future development to contribute to surveillance and diagnosis of, and communication about, arbovirus infections in pregnancy. The findings also indicate that such an app shows promise for future development and implementation by direct treatment and care teams to optimize obstetric care. For any of these potential uses, further research will be required, for example, to explore how app use could be linked to sampling (including self-sampling) and testing within a surveillance program while adapting the mobile app interface, features, and messages to the appropriate cultural context.

## Appendix

Multimedia Appendix 1 Symptoms included in ZIKApp and the start and end of arbovirus episode triggers.

Multimedia Appendix 2 Timing of symptom diary completion, by 30-day period from app initiation.

Multimedia Appendix 3 Type and duration of symptoms reported by women with arbovirus trigger episodes.

## Abbreviations

CHIKV: chikungunya virus
DENV: dengue virus
mHealth: mobile health
REDCap: Research Electronic Data Capture
SOP: standard operating procedure
UHWI: University Hospital of the West Indies
ZIKV: Zika virus
