# Supplementary material for: Detection of Potential Arbovirus Infections and Pregnancy Complications in Pregnant Women in Jamaica Using a Smartphone App (ZIKApp): Pilot Evaluation Study
Source: JMIR Form Res. 2022 Jul 27;6(7):e34423. doi: 10.2196/34423 (PMC9377438; doi:10.2196/34423)
Supplement: Multimedia Appendix 1 [file formative_v6i7e34423_app1.docx]

**Multimedia Appendix 1. Symptoms included in ZIKApp and the start and end of arbovirus episode triggers.**

The diary includes the following signs and symptoms divided in categories based on their specificity for arbovirus infections:

| **Diary items** | |
| --- | --- |
| **Symptom** | **Category** |
| Fever |  |
| Unwell/less fit | A |
| Headache | A |
| Red eyes | B |
| Joint pain | B |
| Muscle ache | A |
| Rash | B |
| Gum, nose or rectal bleeding | B |
| Cold symptoms/cough | C |
| Diarrhoea |  |
| Vomiting |  |
| Painful uterine contractions | D |
| Vaginal bleeding | D |
| **No symptoms** |  |

The following combinations of symptoms trigger as ‘possible arbovirus infection’

| **Start of Episode Triggers** | |
| --- | --- |
| **Trigger type** | **Possible Arbovirus Infection** |
| **Option 1: fever and one B symptom on day 1** | Joint pain |
|  | Rash |
|  | Gum, nose or rectal bleeding |
|  | Red eyes |
| **Option 2: fever and at least two A symptoms on day 1** | Headache |
|  | Muscle ache |
|  | Unwell/less fit |
| **Option 3: no fever, but three out of four of the following symptoms present during at least one of two consecutive days** | Rash |
|  | Red eyes |
|  | Joint pain |
|  | Gum, nose or rectal bleeding |
| **Trigger type** | **Possible Pregnancy complication** |
| **Option 1: one D symptom on day 1** | Painful uterine contractions |
| **Option 2: one D symptom on day 1** | Vaginal bleeding |
| **Trigger type** | **other symptoms that require medical attention** |
| **Option 1: fever on day 1 and 2** | Fever |
| **Option 2: Diarrhoea and/or vomiting on day 1, 2 and 3** | Diarrhoea |
|  | Vomiting |

| **End of Episode Triggers** | |
| --- | --- |
| **Trigger type** | **Possible Arbovirus Infection** |
| **Option 1 and 2:** | reset trigger after 5 days |
| **Option 3:** | reset trigger after 7 days |
| **Trigger type** | **Possible Pregnancy complication** |
| **Option 1:** | reset trigger after 3 days |
| **Option 2:** | reset trigger after 3 days |
| **Trigger type** | **other symptoms that require medical attention** |
| **Option 1:** | reset trigger after 5 days |
| **Option 2:** | reset trigger after 5 days |
