# Supplementary material for: Detection of Potential Arbovirus Infections and Pregnancy Complications in Pregnant Women in Jamaica Using a Smartphone App (ZIKApp): Pilot Evaluation Study
Source: JMIR Form Res. 2022 Jul 27;6(7):e34423. doi: 10.2196/34423 (PMC9377438; doi:10.2196/34423)
Supplement: Multimedia Appendix 2 [file formative_v6i7e34423_app2.docx]

**Multimedia Appendix 2.** **Timing of symptom diary completion, by 30 day period from App initiation**

| **Day since App initiation** | **0-30** | **31-60** | **61-90** | **91-120** | **121-150** | **151-180** | **181-210** |
| --- | --- | --- | --- | --- | --- | --- | --- |
|  |  |  |  |  |  |  |  |
| **Number of women contributing to period** | 157 | 155 | 148 | 131 | 118 | 86 | 26 |
|  |  |  |  |  |  |  |  |
| **Diary completed same day** | 1736 | 1,480 | 1,348 | 1,217 | 1,024 | 440 | 56 |
| **Diary completed 2-3 days later** | 1296 | 1,440 | 1,343 | 1,207 | 932 | 476 | 70 |
| **Diary completed 4-7 days later** | 811 | 816 | 733 | 657 | 500 | 252 | 47 |
| **Not completed (>7 days elapsed)** | 1,014 | 815 | 817 | 674 | 728 | 543 | 163 |
|  |  |  |  |  |  |  |  |
| **Total number of diary days** | 4,857 | 4,551 | 4,241 | 3,755 | 3,184 | 1,711 | 336 |
