# Supplementary material for: Detection of Potential Arbovirus Infections and Pregnancy Complications in Pregnant Women in Jamaica Using a Smartphone App (ZIKApp): Pilot Evaluation Study
Source: JMIR Form Res. 2022 Jul 27;6(7):e34423. doi: 10.2196/34423 (PMC9377438; doi:10.2196/34423)
Supplement: Multimedia Appendix 3 [file formative_v6i7e34423_app3.docx]

**Multimedia Appendix 3. Type and duration of symptoms reported by women with arbovirus trigger episodes.**


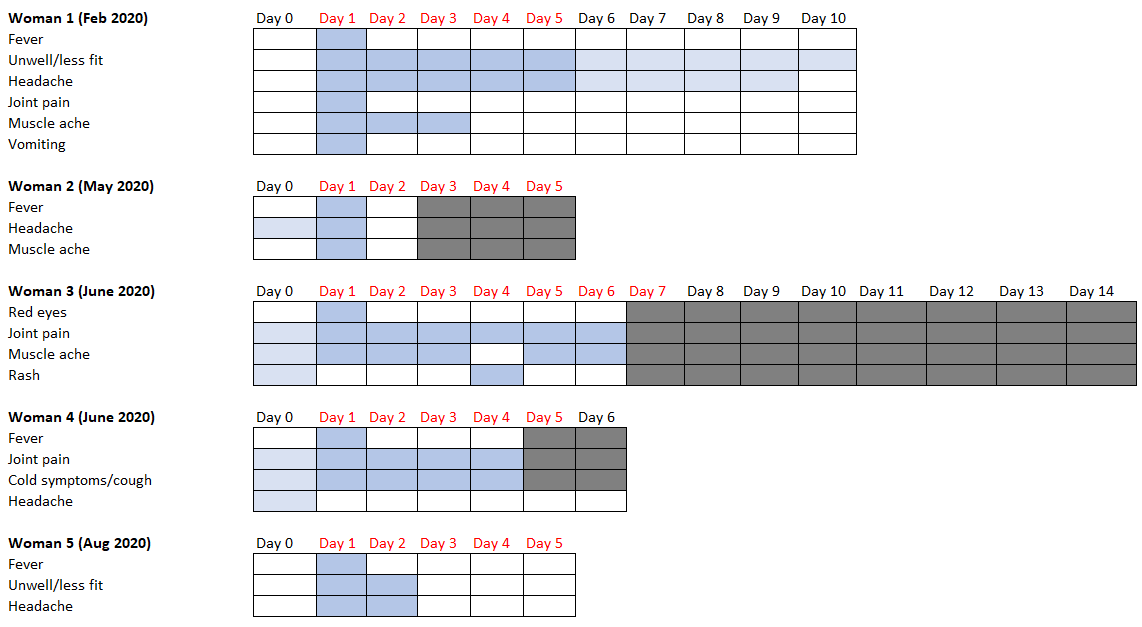


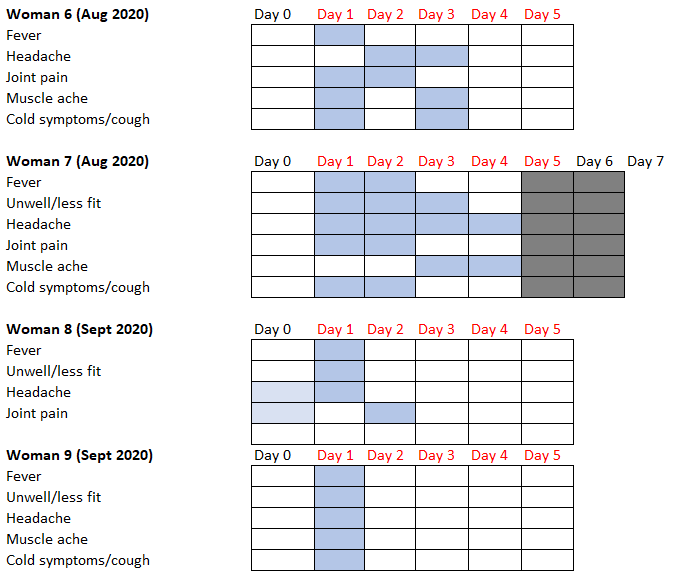


Legend

| Red writing | Days included in the arbovirus trigger episode | Black writing | Days outside of the arbovirus trigger episode |
| --- | --- | --- | --- |
| Unshaded block | Diary completed and no symptom reported |  | Symptom reported within the arbovirus trigger episode |
|  | Symptom reported outside of the arbovirus trigger episode |  | Diary not completed |
